# Long-Term Effects of Self-Control on Alcohol Use and Sexual Behavior among Urban Minority Young Women

**DOI:** 10.3390/ijerph9010001

**Published:** 2011-12-23

**Authors:** Kenneth W. Griffin, Lawrence M. Scheier, Bianca Acevedo, Jerry L. Grenard, Gilbert J. Botvin

**Affiliations:** 1 Department of Public Health, Weill Cornell Medical College, New York, NY 10065, USA; Email: bia2006@med.cornell.edu (B.A.); gjbotvin@med.cornell.edu (G.J.B.); 2 LARS Research Institute, Las Vegas, NV 89135, USA; Email: scheier@cox.net; 3 School of Community & Global Health, Claremont Graduate University, Claremont, CA 91773, USA; Email: jerry.grenard@cgu.edu

**Keywords:** alcohol, sexual behavior, young adulthood, self-control, minority, latent class analysis

## Abstract

High risk alcohol use and sexual behaviors peak in young adulthood and often occur in the same individuals. Alcohol use has been found to impair decision-making and contribute to high risk sexual activity. However, the association between alcohol use and risky sexual behavior may also reflect enduring individual differences in risk taking, sociability, self-control, and related variables. Both behaviors can serve similar functions related to recreation, interpersonal connection, and the pursuit of excitement or pleasure. The present study examined the extent to which high risk drinking and sexual behavior clustered together in a sample of urban minority young adult women, a demographic group at elevated risk for negative outcomes related to sexual health. We tested whether psychosocial functioning measured at the beginning of high school predicted classes of risk behaviors when girls were tracked longitudinally into young adulthood. Latent class analysis indicated three distinct profiles based on high risk drinking and sexual behavior (*i.e.*, multiple sex partners) in young adulthood. The largest class (73% of the sample) reported low levels of risky drinking and sexual behavior. The next largest class (19%) reported high risk drinking and low risk sexual behavior, and the smallest class (8%) reported high levels of both behaviors. Compared to women from other racial/ethnic groups, black women were more likely to be categorized in the high risk drinking/low risk sex class. Multinomial logistic regression indicated that self-control in adolescence had a broad and enduring protective effect on risk behaviors eight years later and was associated with a greater probability of being in the low risk drinking/low risk sex class. Findings are discussed in terms of understanding the phenotypic expressions of risk behavior as they relate to early psychosocial development and the long-term protective function of self-control in reducing high risk drinking and sexual behaviors.

## 1. Introduction

Prevalence of high risk drinking and high risk sexual behaviors peak during late adolescence and young adulthood. In 2010, epidemiological evidence from the National Survey on Drug Use and Health [1] revealed that alcohol use was highest among young adults from 21 to 25 years of age, with 45.5% of these individuals reporting binge drinking (five or more drinks on the same occasion) and 18% reporting heavy drinking (binge drinking on 5 or more days) in the past month. Alcohol abuse among young adults contributes a variety of negative outcomes such as unintentional injuries and deaths, traffic fatalities, sexual assault, academic failure, interpersonal aggression, and psychiatric problems [2,3,4,5]. High risk sexual behaviors and the resulting health effects also disproportionately affect young people. Almost half of all new sexually transmitted infections (STIs) in the US occur among 15 to 24 year olds [6], and half of all new HIV infections in the US occur in young people aged 24 years or under [7].

### 1.1. Co-Morbidity of High Risk Drinking and Sexual Behavior

Research has shown that patterns of high risk alcohol use and sexual behavior often co-occur in the same individuals. Several studies have shown that alcohol abuse is associated with an increased risk for unwanted pregnancy and sexually transmitted infections [8,9]. However, research also shows that the relationship between the two behaviors is complex. Studies have examined patterns of alcohol use and sexual behavior either at the level of *global overlap* or *situational overlap* [10]. Global overlap is the extent to which an individual engaging in one behavior is more likely to engage in another behavior [11]. Studies assessing global overlap generally report positive associations between indices of drinking, sexual frequency, and high risk sex [12,13,14]. *Situational overlap* is a more refined level of analysis that examines whether an individual engaging in one behavior on a specific occasion is more likely to engage in another behavior on the same occasion. Findings from situation or event-level studies show a more nuanced relationship between alcohol use and risky sex. In a comprehensive review on drinking and risky sexual behavior among college students, Cooper [8] distinguished between *indiscriminate sex behaviors* (e.g., casual, non-steady, multiple, or unknown partners, failure to discuss risk topics prior to intercourse) or *failure to take protective actions* (e.g., lack of condom use or birth control). Event-level studies have shown that alcohol can affect indiscriminate high risk sexual behaviors, particularly sex with non-steady partners [14], but its ability to predict failure to take protective action is more complex and conditional.

Conceptual models linking alcohol use and sexual behaviors emphasize the role of *perceived norms*, *expectancies* regarding drinking and sex, *cognitive impairment* due to alcohol consumption, and *motives* surrounding drinking and sex [15,16,17,18,19]. Models of *perceived norms* propose that the extent to which an individual engages in high risk drinking and/or sex is a function of their perceptions of the prevalence of the behavior among people in general or people like them in particular. Individuals who believe that alcohol use prior to sex is common or normative will be more likely to engage in these behaviors compared to individuals who don’t share these perceptions. An *expectancies* approach emphasizes the importance of what an individual expects to happen as a result of drinking (e.g., perceived benefits/costs). In a value-expectancy framework, when perceived positive outcomes outweigh negative ones, this provides an impetus for greater behavioral involvement (more drinking before sex). A *cognitive impairment* model suggests that alcohol-induced deficits in cognitive functioning limit one’s ability to evaluate risks, such that risks associated with sex are less likely to be perceived, encoded, and fully processed when intoxicated. A *drinking motives* model focuses on the psychological functions of alcohol consumption for an individual in a particular context. For example, an individual may observe that their own alcohol consumption facilitates social interaction that may lead to sex, and thus drink as a way to disinhibit their behavior and reduce feelings of awkwardness in social situations.

This brief review demonstrates that both empirical research and psychosocial theory have focused extensively on the links between high risk drinking and sexual behaviors. However, the association between these behaviors may be spurious to the extent that both are manifestations of long-standing individual differences in risk-taking propensity, social confidence, self-control, and related variables [14,20,21]. Alcohol use and sexual activity both typically involve social activity and may serve similar functions related to recreation, relaxation, interpersonal connectedness, and the pursuit of excitement or pleasure. They are both appetitive behaviors associated with an urge to consume in order to satisfy a physical or psychological need. Because of these similar influences and functions, one might expect high risk drinking and sexual behaviors to occur in the same set of individuals or result from similar etiologic processes. 

### 1.2. The Development and Expression of Self-Control

A central component in understanding appetitive behavior is the concept of self-control, which has been defined as the deliberate, conscious, effortful control over behavior, attention, thoughts, emotions, performance, and impulses [22,23,24]. When people exercise self-control they are more deliberative and thoughtful in their decision-making process and thus self-control may contribute independently to decisions to avoid alcohol consumption and high risk sexual behaviors. In fact, research has demonstrated that self-control is a robust protective factor for a number of social and health risk behaviors, particularly during adolescence [25,26]. Conversely, low self-control is significantly associated with a variety of negative outcomes including illicit drug use, obesity, and depressive symptoms [27,28]. Self-control develops out of a complex interaction of biological, psychological, and social factors. Innate factors related to self-control include underlying neural, physiological, and genetic variables as well as temperament [21,29,30]. Social and environmental factors such as caregiving support, sibling and peer relationships, and social norms play an important role in the development of self-control [31,32]. A number of studies have shown that maladaptive family functioning in childhood (e.g., parent mental illness or substance abuse; family violence; physical, emotional, or sexual abuse) predisposes individuals to poor self-control in adulthood [33]. Conversely, factors such as social support, parental monitoring, and appropriate discipline provide a structured social environment that promotes self-control among children and adolescents [34]. Furthermore, social and environmental factors may interact with genetic influences to produce low self-control [35].

Wills and colleagues have described how temperament factors that develop in early childhood predict later self-control and substance use in adolescence [21]. According to their conceptual model, temperament factors such as the propensity for activity, emotionality, attention, sociability, and inhibition provide a substrate for the later development of self-control during later childhood and adolescence. Because temperament reflects the *style* of typical behavior for an individual, rather than its *content*, the effect of temperament on the development of self-control and engagement in risk behavior is shaped by a combination of socializing agents in one’s environment (e.g., parents, teachers and peers) and individual-level psychosocial risk and protective factors. As individuals mature in cognitive and social skills during later childhood and adolescence, temperamental characteristics result in phenotypic expressions of complex self-control skills such as goal-setting, impulse control, and delay of gratification. These regulatory functions, in combination with environmental and social factors, specify how self-control is exhibited and whether it is protective regarding risky behavior [36,37]. For example, low self-control may be linked to the onset or escalation of substance use by increasing vulnerability to negative life events, deviant peers, or poor academic competence [26,38,39,40]. 

### 1.3. A Focus on Urban Minority Women

National surveillance data show that patterns of risk behavior differ across demographic subgroups of adolescents and young adults. Much of the research focus on alcohol use among young adults has been on college students because the frequency and quantity of alcohol consumption among those who attend college typically surpass rates among nonstudents during the college years [41,42]. College students have also been the focus of a substantial amount of research on the patterns and health consequences of high risk sexual behavior [43]. Studies of college student populations are limited in that many often focus on fairly homogenous samples of middle class, white youth. There is a need for more research on the epidemiology, etiology, and consequences of high risk drinking and sex among other demographic groups. Indeed, research has documented consistently higher rates of teen pregnancy, HIV and other STIs among racial/ethnic minority groups in the US compared to whites [44,45]. A recent CDC report [46] revealed that 46% of people diagnosed with HIV in the US in 2009 were black/African American and that high-risk heterosexual contact among women accounted for more than half of HIV/AIDS cases. Among women diagnosed with AIDS in the US in 2009, 78% were black/African American. Thus, it is important to examine individual-level psychosocial factors among urban minority women in order to increase our understanding of the causes of high risk behaviors and to inform the development of more effective preventive interventions for this group. 

### 1.4. Study Goals

The goals of the present study were to examine whether there are distinct classes of high risk drinking and high risk sex among urban minority young adult women, a demographic group at high risk for negative outcomes related to sexual health. We sought to determine the extent to which high risk drinking and high risk sexual behavior cluster together or occur independently of one another in this population. Further, we examined whether several domains of psychosocial functioning potentially relevant for both behaviors (*i.e.*, self-control, risk taking, social confidence, and self-esteem) were efficient predictors of class membership over a period of time from the beginning of high school to young adulthood. If adolescent risk and protective factors contribute in a similar fashion to high risk drinking and sexual behavior, it may help explain why these two behaviors cluster together among some individuals. 

## 2. Methods

### 2.1. Sample

Participants in the present study were part of a larger school-based drug abuse and violence prevention trial conducted with a sample of predominantly minority urban youth. For the present analysis, we included girls from schools that were randomized to the control group condition in order to examine developmental hypotheses in the absence of intervention. Participants were included in the analysis if they were present in the 9th grade and also present at the last follow-up assessment conducted in young adulthood (N = 692). The 9th grade sample included girls (N = 1,233), with a mean age of 14.6 (SD = 0.47), from 21 New York City public and parochial middle schools. Approximately 46% of participants received free lunch at school, 31% lived in mother-only households, and 48% lived with both parents. The racial/ethnic composition was 54% black, 21% Hispanic, 5% white, 5% Asian, 4% American Indian, and 11% reported other or mixed racial self-identification. 

At the time of the young adult follow-up interview, 38% of participants reported being single, 39% had a girlfriend/boyfriend, 15% were in some type of committed marital or significant partner relationship, and 8% were engaged. The mean age of participants at the young adult assessment was 22.8 years (range 21–26). Almost three-quarters of the sample (70%) reported not having any children, 21% had one child, and 9% had two or more children. Less than 11% of the sample had not received a high school diploma, 53% had received a high school diploma or equivalent, 12% had an associate degree, and 24% had a bachelor’s degree or higher. In terms of occupational status, 37% said they were employed part-time, 35% said they had one full-time job, and 28% said they were not employed. About one third of participants (34%) indicated that they earned less than $5,000 a year, 21% earned between $5,000 and %10,000, 18% between $10,000 and $20,000, 14% between $20,000 and $30,000, and 9% reported income of over $30,000 (3.7% did not answer this question).

### 2.2. Procedure

In the 9th grade, students completed a self-report questionnaire that assessed a wide range of substance use behaviors (*i.e.*, alcohol, cigarettes, marijuana, and inhalants) and psychosocial variables. Unique identification numbers were pre-coded onto each survey rather than student names in order to ensure confidentiality, and each pre-coded survey was distributed to the appropriate student by trained data collectors. These unique ID codes were used to match student surveys over the course of the study, using a master list connecting the IDs to the student’s name that was kept under lock and key by the investigator. Students were informed that their responses would not be made available to school personnel, teachers, or parents. Questionnaires were administered during regular classroom periods over a two-day period by a team of several data collectors, and all students completed the same version of the questionnaire. 

Participants were followed-up once as young adults in 2009 to 2010 and were mailed a packet of information requesting their participation in a telephone survey. The packet included a brief description of the continuing study, a 90-day calendar to be used during a scheduled telephone interview, along with $2 and an offer of additional compensation ($40 to $60) upon completion of a phone interview. Participants were provided with a toll-free telephone number in order to complete the interview. During the telephone interview, participants were provided with a brief description of the study and were asked for oral consent to participate in a telephone interview which included questions about their alcohol and drug use, history of sexual partners, participation in high risk sexual behavior, a variety of psychosocial measures, and personal descriptive information (*i.e.*, income, employment, occupation, marital history, and other demographic items). A portion of the interview assessed risk behaviors over the past 90 days using timeline follow-back (TLFB) interview procedures. The TLFB interview method is a widely used assessment procedure that uses calendar-based guided recall to accurately measure alcohol and illicit drug use [47] or HIV risk behavior [48]. TLFB procedures have been shown to be psychometrically valid when completed either in person or over the telephone [49]. As a first step in this procedure, a trained interviewer reviewed a 90-day calendar with the participant, marking on the calendar holidays and significant events (birthdays, anniversaries, illnesses of oneself or close friends and family, or any event that was deemed personally meaningful to the participant) to create “anchor points” for facilitating accurate recall. The interviewer then guided the participant to elicit their day-to-day risk behaviors working backwards over a 90-day period. The length of the telephone interview depended on the degree of engagement in risky behavior and ranged from 20 minutes for respondents engaging in little risk behavior to over 60 minutes for individuals engaging in frequent risk behavior. The research protocol and consent procedures were reviewed and approved by the Institutional Review Board at Cornell Medical College.

### 2.3. Measures

Young Adult Latent Class Indicators of Risk Behavior. All of the indicators for the Latent Class Analysis (LCA) models were categorized *a priori* to indicate low and high risk. The alcohol use items included frequency of drinking beer, wine, or hard liquor (a few times a month *vs.* once a week), frequency of drinking until you get drunk (not getting drunk *vs.* a few times per year), quantity of drinks per drinking occasion (three or less *vs.* four or more), and number of days having five or more drinks (no days *vs.* one or more days). The same procedure was used for the high risk sexual behavior items, with four items including number of sexual partners in the past three months (one or less *vs.* two or more, respectively), a quantified index of partners (one or less sets of initials used by respondent to indicate sexual activity *vs.* more two or more sets of initials), whether or not the participant had a new partner within the past three months, and number of sex event with individuals not designated as main partner (one *vs.* two or more sexual events).

Adolescent Psychosocial Markers. The following measures were included at the 9th grade assessment and were used to predict membership in the latent classes at the young adult follow-up. The variables reflect individual-level characteristics that may instigate high risk drinking and sex (propensity for risk taking) and social and interpersonal factors that either facilitate (social confidence and self-esteem) or inhibit engagement in these behaviors (self-control).

*Risk-Taking*. Four items (α = 0.80) were taken from the Eysenck Personality Inventory [50] to assess impulsive and daring behavior. Sample items include “I would do almost anything on a dare” and “I enjoy taking risks.” Students indicated responses on a 5-point scale ranging from (1) *really not true for me* to (5) *really true for me*.

*Social-Confidence*. A 14-item scale assessed students’ confidence about their ability to use specific social skills (α = 0.82). Participants were provided a stem “How confident are you that you could do well in the following situations...” with sample items including “ending a conversation with friends without offending them,” “starting a conversation with someone you’ve just met,” and “asking questions to avoid a misunderstanding.” Response categories ranged from (1) *not at all confident* to (5) *very confident*. 

*Self-Esteem*. Five items from the [51] Self-Esteem Scale were used to assess the positive evaluative component of self-esteem (α = 0.85). Sample items include “I feel that I have a number of good qualities,” and “I have a positive attitude about myself.” Response categories ranged from (1) *strongly disagree* to (5) *strongly agree*.

*Self-Control*. Fourteen items (α = 0.72) from the Kendall and Wilcox Self-Control Rating Scale [52] were used to measure self-control skills. The SCRS assesses cognitive-behavioral elements of self-regulation including the ability to manage impulsive, distracting, or disruptive behavior, particularly those occurring in school settings. Sample items include “I stick to what I am doing until I’m finished with it,” “I have to be reminded several times to do something,” and “I am easily distracted from my work.” Response categories ranged from (1) *strongly disagree* to (5) *strongly agree*. 

### 2.4. Data Analysis Plan

Latent class analysis (LCA) represents a broad class of random coefficient analytic methods that can be used to summarize meaningful subgroups of individuals based on their patterns of response profiles. In recent years, investigators using LCA have been successful in defining unique classes of individuals based on nicotine use [53], patterns of delinquency [54], problem behaviors [55], and different types of drug use [56,57,58]. This approach has become an important alternative to traditional variable-centered methods that can produce summaries of how one variable relates to another (*i.e.*, traditional factor analysis methods) but provides little information as to how individuals cluster with regard to their response profiles. In the simplest case with two questions each having yes/no answers, a 2 × 2 cross-tabulation produces four possible response profiles (including yes-yes, yes-no, no-no, and no-yes). When the possible contingency table is this straightforward the naked eye can scan the possible response profiles and an investigator can apply the chi-square statistic to assess the degree of dependence among measures. However, when the sheer number of possible profiles expands considerably (*i.e.*, with 8 dichotomous items there are 2^8^ or 256 possible response patterns), more refined statistical methods that can consider probabilistically the joint probabilities of a multiway table are needed to identify meaningful response patterns [59].

LCA is traditionally employed with categorical indicators (*i.e.*, yes/no) that are used to define membership in discrete groups. These indicators are considered imperfect, but informative with regard to a latent (unobserved) categorical variable that contains discrete and mutually exclusive subpopulations or classes. The members of a particular class differ from each other only with respect to random measurement error but are uniquely and systematically different from members in other classes based on their response profiles [60]. Important model parameters include the latent class prevalence or proportion of individuals assigned to a particular class (*i.e.*, estimated posterior probabilities) and the item response probabilities, which are measurement parameters interpreted as the likelihood of endorsing a particular item if you are a member of a particular class. As we explain below, statistical fit can be evaluated on the basis of how well a particular model positing a latent categorical variable with *k* classes can reproduce the observed response patterns (observed *vs.* expected cell frequencies) and correctly classify individuals (*i.e.*, the model produces consistent and efficient estimators), the parsimony achieved through a reduced number of classes, whether an obtained latent class model is theoretically interesting, and the overall stability of the obtained classes.

## 3. Results

### 3.1. Attrition Analyses

There was 56% sample retention from the 9th grade to the young adult assessment. Mean comparisons showed that participants retained through young adulthood did not differ significantly from dropouts on their alcohol consumption (measured by a mean composite of drinking frequency, intensity, and drunkenness). A logistic regression model indicated that several demographic measures predicted attrition in the study, including being older in age (b = 0.381 [SE = 0.179], being black (b = 0.433 [SE = 0.168], and coming from a single parent home (b = 0.589 [SE = 0.168], all *p* < 0.05. This suggests that those who dropped out of the study were at higher risk than those who remained in the study. 

### 3.2. Prevalence of Young Adult High-Risk Behaviors

Prevalence estimates for the latent variable indicators showed that 13% of the young adult sample reported drinking at least once a week, 31% reported drinking until they got drunk, 17% said they were having at least four or more drinks each time they drank, and 26% reported heavy drinking (one or more days where they drank five or more drinks per occasion). Regarding the sexual risk items, 8% of the panel sample respondents said they had two or more sexual partners in the past three months, an additional 8% provided two or more sets of initials for sexual partners spanning the same period, 18% said the individual they had sexual relations within the past three months was a “new” partner, and of those who had multiple partners 28% reported multiple sex events with someone other than their main partner. We used the MCMC procedure for arbitrary missing data under the assumption of missing at random. This procedure relied on single imputation for these analyses (there was no gain in efficiency for estimating the standard errors with more than a single imputation).

### 3.3. LCA Results

Table 1 shows the fit statistics corresponding to the sequence of models tested. Models were tested from the most parsimonious one class model (all participants responded in the same way) to a solution with ten classes. Evaluative model fit statistics include the Bayesian Information Criteria [61] and the Akaike’s Information Criteria [62,63], both of which penalize the log-likelihood for increased number of parameters as models increase in complexity (*i.e.*, increasing the number of classes). The log likelihood ratio test statistic (L^2^) shows the amount of variation left in the model among the variables after extracting the classes (in all cases smaller numbers indicate a better fit). The likelihood ratio chi-square test statistic (G^2^), which assesses “departure” of the population or expected model from the observed sample data, should be evaluated with respect to the degrees of freedom in the model (an approximate value of 1.0 indicates better fit). A well fitting model will have G^2^ distributed as a chi-square statistic with the degrees of freedom equivalent to the number of possible response patterns (in the current model this is 2^8^) less the number of estimates (indicator variables) minus one [64]. The column labeled L^2^/df provides an approximate F-statistic [65].

**Table 1:** Fit statistics from the latent class analyses.

Number of classes	Log-likelihood (L^2^)	BIC-L^2^	AIC-L^2^	Npar/DF	G^2^	L^2^/df	*p*-value ^a^	%ER^b^
1-class	2548.2	5148.7	5112.4	8/238	880.3	10.71	0.0000	0.000
2-class	2216.3	4543.7	4466.6	17/238	472.1	9.31	0.0000	0.087
3-class	2060.1	4290.3	4172.3	26/229	159.8	8.99	0.9829	0.081
4-class	2038.6	4306.0	4147.1	35/220	116.7	9.26	1.0	0.800
5-class	2022.1	4332.0	4132.2	44/211	83.8	9.58	1.0	0.794
6-class	2014.8	4376.1	4135.5	53/202	69.1	9.97	1.0	0.790
7-class	2009.2	4423.9	4142.4	62/193	57.9	10.41	1.0	0.790
8-class	2005.1	4474.4	4152.1	71/184	49.6	10.89	1.0	0.787
9-class	2001.3	4525.8	4162.7	80/175	42.2	11.44	1.0	0.785
10-class	1997.4	4576.9	4172.8	89/166	34.4	12.03	1.0	0.784

Note: ^a^ Significance values can be computed using the Lo-Mendell-Rubin likelihood-ratio test (Lo *et al.*, 2001) allowing for direct tests between models with ‘*k*’ and ‘*k-1*’ classes. Low p-values indicate the model with one less class should be rejected in favor of the estimated model. ^b^ %ER = percent error reduction in L^2^ when model is pitted against the null model of complete independence (one-class model).

An inspection of Table 1 shows that the AIC and BIC fit indices become progressively smaller through the three and four class models and the error terms in these models was substantially less than models containing more classes. Notably, extraction of the fourth class (low risk drinking, high risk sex) required migration of participants from other classes and the resulting class was quite sparse (N = 33, or 4% of the total sample was located within this class). The low cell counts associated with the four class solution might increase the likelihood of misclassification and strain the robustness of the subsequent estimation procedures. This is consistent with the notion that, in the process of identifying a satisfactory latent class model, one can reach a saturation point that produces weak identifiability [66] beyond which there are too many classes, too few reliable class indicators, and too few people allocated to the classes [67]. With these considerations in mind, the three-class model provided the best fit based on shrinkage in the AIC and BIC, the G^2^, the percent error reduction (an alternative fit to entropy-based measures) and the overall ratio of L^2^/df.

**Table 2:** Comparison of parameters across the unconstrained and constrained models.

	Model 1: Unconstrained			Model 2: Constrained		
	Class 1	Class 2	Class 3	Class 1	Class 2	Class 3
Black Participants						
Proportion	0.033	0.113	0.304	0.033	0.118	0.299
Count (N = 312)	23	78	211	23	82	207
	7%	25%	68%	7%	26%	66%
Item Probabilities (for higher risk responding)						
Drinking Frequency (Alc1)	0.348	0.413	0.046	0.263	0.409	0.046
Drunkenness Frequency (Alc2)	0.652	0.935	0.122	0.526	0.899	0.134
Drinking Quantity (per occasion) (Alc3)	0.522	0.573	0.062	0.316	0.571	0.051
Binge Days in Past 3 Months (Alc4)	0.696	0.760	0.111	0.544	0.766	0.102
# Sexual Partners (Sex1)	1.000	0.000	0.000	1.000	0.000	0.000
Recent Sexual Partners (Sex2)	1.000	0.000	0.000	1.000	0.000	0.000
New Sexual Partners (Sex3)	0.826	0.088	0.086	0.772	0.141	0.120
Sex Events w/ Non-Main Partner (Sex4)	0.913	0.204	0.209	0.912	0.271	0.215
	**Class 1**	**Class 2**	**Class 3**	**Class 1**	**Class 2**	**Class 3**
Other Participants						
Proportion	0.049	0.092	0.407	0.049	0.075	0.425
Count (N = 380)	34	64	282	34	52	294
	9%	17%	74%	9%	14%	77%
Item Probabilities (for higher risk responding)						
Drinking Frequency (Alc1)	0.206	0.371	0.035	0.263	0.409	0.046
Drunkenness Frequency (Alc2)	0.441	0.734	0.133	0.526	0.899	0.134
Drinking Quantity (per occasion) (Alc3)	0.176	0.475	0.038	0.316	0.571	0.051
Binge Days in Past 3 Months (Alc4)	0.441	0.719	0.072	0.544	0.766	0.102
# Sexual Partners (Sex1)	1.000	0.000	0.000	1.000	0.000	0.000
Recent Sexual Partners (Sex2)	1.000	0.000	0.000	1.000	0.000	0.000
New Sexual Partners (Sex3)	0.735	0.281	0.125	0.772	0.141	0.120
Sex Events w/ Non-Main Partner (Sex4)	0.912	0.438	0.195	0.912	0.271	0.215

Notes: Proportions represent classification of individuals based on their most likely latent class pattern; Count represents latent class count based upon most likely class.

Item response (or endorsement) probabilities show that Class 1 (8% of participants) contains women who reported high risk drinking and high risk sexual behaviors (referred to as “high-high”). The second class (19%) contains women who reported high risk drinking but low risk sex (referred to as “high-low”). The third and largest class (73%) contains women who reported low risk drinking and low risk sex (referred to as “low-low”). After deriving the three-class model we estimated a mixture model with race/ethnicity as a grouping variable (black *vs.* other). This model addresses whether race/ethnicity contributes to any of the heterogeneity underlying the classes as well as defining class membership. Table 2 shows the class membership probabilities and item response probabilities for the three class model tabulated by race/ethnicity. Model parameters are presented for both constrained and unconstrained models; the former constrained the item response probabilities across the two race/ethnicity groups. The conditional likelihood ratio difference test and the non-significant p-value indicated the model with these constraints was tenable, ΔG^2^(24) = 34.32, *p* > 0.10 (*i.e.*, there was no degradation in fit by implying the item thresholds and class probabilities are equal across groups). Class assignment probabilities from the unconstrained model indicated that 7% of black women were assigned to the high-high class, 25% were assigned to the high-low class, and 68% were assigned to the low-low class (based on N = 312). The same numbers for the non-black women (based on N = 380) indicated that 9% were assigned to the high-high class 1, 17% were assigned to the high-low class, and 74% were assigned to the low-low class. 

**Figure 1:**
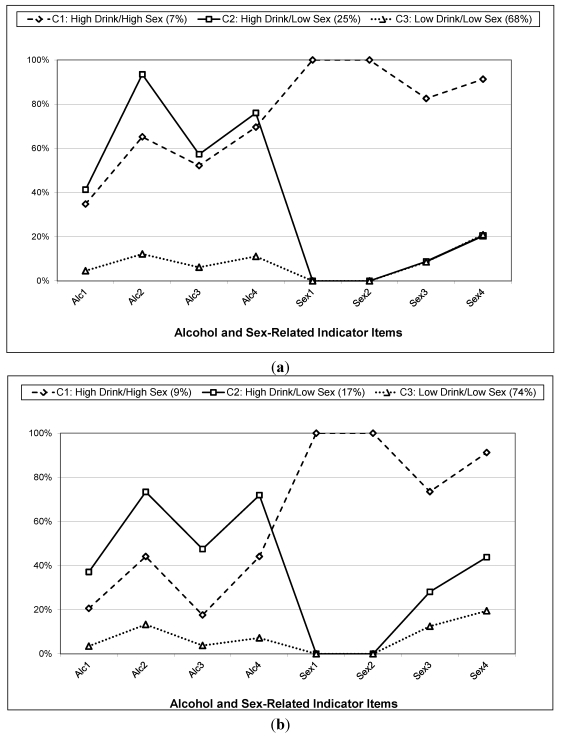
(**a**) Item response probabilities by class membership among black women; (**b**) Item response probabilities by class membership among other women (reference category).

Figure 1(a,b) illustrate the item response probabilities for each alcohol and sex-related indicator for black women and for women of other race/ethnicities. Members of the low-low class did not exceed the critical 0.5 threshold for any single item (this pattern held for black and non-black women). Compared to women from other race/ethnic groups, black women in the low-low class were more likely to report drinking excessively for more than five days in a row and less likely to report new sexual partners in the same time frame. Black women in the high-low class were characterized by high likelihood of drinking to get drunk, episodes of drunkenness, and number of days having five or more drinks but low levels of all high risk sexual behaviors. Black women in the high-high class were characterized by high scores for all four sexual behavior items and drinking until drunk and binge drinking. The same pattern of endorsement was not evident for the non-black women in the high-high class. These individuals were much less likely to report high risk drinking and generally equally likely to report high risk sexual behaviors. 

### 3.4. Results of the MNR Model

After extraction of the classes grouped by race/ethnicity, the next step included examining several possible determinants of class membership using multinomial logistic regression. The reference class for these comparisons was Class 3 (low risk drinking/low risk sex). The MNR model held constant some of the important 9th grade socio-demographic factors that can contribute to class differences including nuclear family status (intact *vs.* other), free lunch (receipt *vs.* no subsidy), and a continuous measures of self-reported grades (a proxy for conventional behavior and school bonding). Other measures of psychosocial functioning modeled included risk-taking, social confidence, self-esteem, and self-control. Table 3 presents the findings from the MNR including regression coefficients and relative odds ratios for class membership based on these external markers. Findings indicated that self-control played an instrumental role for members of Class 1 (high-high). Compared to members of Class 3 (low-low), the logs odds of their being assigned to the high-high class decreased by −0.571 for a unit increase in self-control (*i.e.*, indicating a strong protective role for self-control). Nuclear family status also helped to distinguish members of Class 1 (high-high) from the reference Class 3 (low-low). Residing with both biological parents was also protective as the log odds of being assigned to Class 1 (high-high) decreased −0.611 for a unit increase in family status (*i.e.*, living in an intact family).

**Table 3:** Relative odds of latent class membership.

	Beta		*p*-value		Odds	
					Relative to Class 3	
	Class 1	Class 2	Class 1	Class 2	Class 1	Class 2
Black (Reference = Other)	0.171	0.824	0.540	0.002	1.186	2.280
Psychosocial Markers (9th Grade)						
Risk-Taking	−0.058	0.180	0.712	0.154	0.944	1.197
Social Confidence	0.247	0.108	0.091	0.407	1.280	1.114
Self-Esteem	0.390	−0.197	0.108	0.210	1.477	0.821
Self-Control	−0.571	−0.275	0.002	0.101	0.565	0.760
Covariates (9th Grade)						
Grades In School	0.215	−0.034	0.128	0.818	1.240	0.967
Free School Lunch	0.102	−0.426	0.721	0.094	1.107	0.653
Nuclear Family	−0.611	0.081	0.038	0.731	0.543	1.084

Notes: Class 1 (High Risk Drinking, High Risk Sex); Class 2 (High Risk Drinking, Low Risk Sex); Class 3 (Low Risk Drinking, Low Risk Sex).

**Figure 2:**
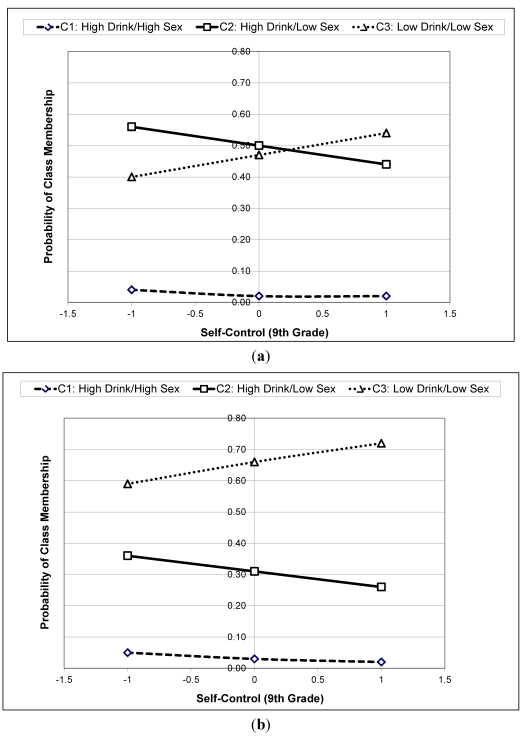
(**a**) Probability of class membership in young adulthood as a function of 9th grade self-control among black women; (**b**) Probability of class membership in young adulthood as a function of 9^th^ grade self-control among other women (reference category).

Figure 2(a,b) graphically portray the association between 9th grade self-control and class assignment for the different race groups. Figure 2(a) shows that for black women self-control plays a minimal role in the risk of assignment to Class 1 (high-high); the line is relatively flat and unchanging for unit increases in self-control. However, the probability of assignment to Class 3 (low-low) increases substantially with corresponding increases in self-control (*i.e.*, protective effect). The gross differences in these two slopes is what contributes to the significant effect shown in Table 3 with the corresponding negative beta weight (b = −0.571) for Class 1 versus Class 3. Although the difference between Class 1 and Class 2 was not significant, the probability of assignment to Class 2, designated the high-low risk group (characterized by excessive drinking practices), decreases with corresponding increases in self-control (these individuals can potentially migrate to the other two classes).

Figure 2(b) shows the same plots for the non-black women in the sample. Here, the effects also shows that self-control is protective for members of Class 3 (low-low) in ways that do not appear to apply for members of Class 2 (high-low) in that self-control decreases; and Class 3 (low-low) with a relatively flat line. There are also some notable differences between the plots. Although the effect of self-control is fairly consistent for women of both black and non-black race groups assigned to the high-high risk group (relatively flat), the slope of the line for the low-low risk group is much steeper for the non-black race group showing that self-control is more protective (increasing levels of self-control increases the odds of being assigned to this group). Also notable is that probabilities for class assignment for Class 2 (high-low) are somewhat higher in the black compared to non-black women, although the line is negatively sloped in both groups. 

## 4. Discussion

The present study examined the extent to which high risk drinking and sexual behavior clustered together in a sample of urban minority young adult women, a population that has been shown to be at elevated risk for HIV, STIs, and other negative outcomes related to sexual health. We also examined whether several relevant aspects of psychosocial functioning measured at the beginning of high school predicted classes of risk behavior years later when participants were assessed as young women. Young adulthood provides a unique window to examine high risk drinking and sexual behavior. These behaviors peak during the early to mid-twenties, as young people live more independently and autonomously from their family of origin, enjoy new freedoms such as legal drinking and the ability to enter bars and nightclubs, and have increased opportunities for sexual and romantic relationships. Indeed, *emerging adulthood* provides young adults with the opportunity to pursue novel, intense, and risky experiences with greater freedom compared to any other developmental period [68]. 

Because alcohol use and sexual behavior share a number of similar social and personal functions, one might expect widespread comorbidity for engagement in high risk levels of these behaviors in young adulthood. Drinking and sexual activity are both appetitive behaviors that young people may pursue for the purposes of recreation, relaxation, social connection, excitement, and/or pleasure. Furthermore, both behaviors may be influenced similarly by individual-level variables that promote engagement in behavior (e.g., propensity for risk taking), social and interpersonal factors that facilitate behavior (e.g., social confidence and self-esteem), and characteristics that inhibit engagement (e.g., self-control) [20,21]. However, findings from the present study showed that overlap between high risk drinking and sexual behavior in the same individuals was relatively infrequent. At the young adult assessment point, there were three latent classes or homogenous groups of women based on their patterns of high risk alcohol use and sexual behaviors. The largest class (73%) represented women characterized by low risk alcohol use and low risk sexual behaviors (“low-low”). The next largest class of women (19%) was characterized by high risk alcohol use and low risk sexual behaviors (“high-low”). The smallest class (8%) was characterized by high risk alcohol and high risk sexual behaviors (“high-high”). Thus, in the final three class model, we found a low proportion of individuals in the “high-high” class, and over twice as many women in the “high-low” class (high risk drinking/low risk sex). In choosing the three over the four class solution, we noted the relatively small proportion of individuals (4%) assigned to a low risk drinking and high risk sexual behavior class; that migration from other classes likely created this small class of individuals; and that using a four class solution would likely create estimation problems given the sparse cells.

Although there was less overlap in risk behaviors than expected, we found that both risky drinking and risky sexual behaviors were predicted in a similar manner by early adolescent psychosocial functioning. In particular, self-control measured at 14 years of age—but not risk-taking, social confidence, or self-esteem—reduced the likelihood of being in the high-high class eight years later when the girls were young adults. This demonstrates a broad and enduring protective effect of self-control on multiple high risk behaviors over a lengthy period of developmental change. These findings provide evidence that the benefits of self-control persist into young adulthood and are not limited to children and adolescents, who have been the focus of most research in this area [25,36,69,70]. 

### 4.1. Implications for Self-Control and Addictive Behaviors

Although the focus of this paper was on high risk drinking and sex, our findings may have implications for compulsive or addictive patterns of alcohol abuse and sexual behavior. In particular, they may be informative regarding addiction specificity, or the extent which an individual engages in one category of addictive behaviors but not another. Sussman *et al.* [71] propose a model of addiction specificity that outlines how a number of biological, environmental, situational, and learning factors may explain why some addictive behaviors occur in an individual at the exclusion of other seemingly similar behaviors. Our findings of relatively low levels of overlap in patterns of risky drinking and risky sex are consistent with the idea of addiction specificity: the two behaviors were found to co-occur in a relatively small proportion of the sample even though individuals may engage in both of these behaviors for similar purposes. 

Self-control is central to the concept of addiction. Someone engaging in an addictive behavior does so compulsively and experiences a subjective inability to control consumption or stop behavior when it has clear negative consequences. Indeed, there are a number of similarities between conceptual models of self-control [21] and the model of addiction specificity [71]. Both describe multifactorial etiologic mechanisms resulting from a complex interplay of biopsychosocial influences that may include factors such as genetic and neurobiological systems, as well as exposure to unique environments, experiences, and learning opportunities, that can begin to influence behavior or precursors of behavior in childhood and adolescence. Also for both self-control and addiction specificity, their ultimate expression is shaped by a variety of socialization factors and environmental influences that provide opportunities for engagement and social reinforcement. Thus, the development of high risk and compulsive behaviors, as well as the ability to control these behaviors, is a function of a variety of contingencies that lead to different trajectories of behavior. These may channel specific individuals towards either high risk drinking or risky sexual behavior. The low levels of overlap between these behaviors in our study may reflect the large number of variables that can contribute to individual trajectories, resulting in differential expression of high risk drinking and sexual behaviors. 

Theory and research suggest that self-control can have direct and indirect effects on risk behavior. For example, Wills and Dishion [72] describe how self-control stems from early temperamental factors including attentional focusing, a characteristic that contribute to adaptive problem-solving and enhance self-control abilities in ways that provide direct protective effects. Alternatively, self-control can also be protective through indirect or buffering effects. It may, for example, buffer the impact of negative peer influences such that teens with good self-control would be less influenced by substance using peers. Empirical evidence also shows that self-control and similar variables related to self-regulation can have direct and indirect protective effects on risky drinking and risky sex. Quinn and Fromme [20] found that self-regulation served as a protective factor against risky drinking and sexual behavior in a sample of college students over the age of 21; they found that self-regulation was particularly important for buffering the impact of heavy episodic drinking on unprotected sex among participants high in risk-taking. Although we did not test for buffering effects of self-control in our analyses, we did find a durable and direct protective of effect of self-control on risky drinking and sex over a lengthy time period of developmental change. Further, the protective effect of self-control remained significant when other key variables (risk taking, social confidence, and self-esteem) that may be correlated with self-control and/or the outcomes were controlled for in the model. 

To the extent that self-control has enduring protective effects across multiple risk behaviors during the transition to young adulthood, implementing effective interventions to increase self-control would serve to promote healthy behavior and enhance public health. Dual process conceptual models suggest that impulsivity and self-control work together to predict engagement in high risk behavior. People balance their impulses and urges for immediate gratification with behavioral self-restraint in order to achieve longer term goals or to act in accordance with personal standards, attitudes, or expectancies [73]. Experimental studies suggest that interventions may focus on factors associated with impulsive tendencies, such as efforts to modify automatic maladaptive associations linking an unhealthy behavior to positive affect, or training individuals to direct attention away from unwanted temptation [74]. However, more research has focused on interventions to increase self-control abilities. In this context, self-control is often conceptualized as a limited resource that can be depleted with use—much like a muscle that becomes tired after exertion. To further the muscle analogy, it appears that self-control can be strengthened by regular “exercise.” Several studies have shown that people can enhance their self-control abilities such that their “strength” is less quickly depleted when responding to demands [75]. 

### 4.2. Strengths and Limitations

Strengths of the study included the longitudinal panel data collected over a period from adolescence to young adulthood in a sample of ethnic minority young women from economically disadvantaged urban communities. The study utilized a unique person-centered data analysis approach relying on latent class analysis to examine subgroup heterogeneity that might otherwise have escaped detection using conventional data analytic methods. This method provides an alternative way to summarize behavior by sifting through categorical response profiles and capturing similarities in the way participants answer questions about their behavior. Other data analysis approaches may have missed the subtle nature of the underlying classes and as a result also missed the protective effects self-control exerts on high risk behaviors.

Several limitations should also be noted. The study utilized a restricted range of sexual behavior measures which focused largely on the number of partners and frequency of sexual activity. These are only a subset of the spectrum of variables that relate to high risk sexual behavior and have relevance for sexual addiction. This may explain in part the lack of a low drink/high sex group from the LCA analysis. There was substantial participant attrition between the 9th grade and the young adult follow-up, thus findings may have limited generalizability to the most at-risk members of this population. The study took a cross-sectional “snapshot” of the lives of these individuals at one point in time as they entered young adulthood. Of course, many events transpired between adolescence and the follow-up interview conducted in young adulthood that may have influenced engagement in high risk drinking and sex. Nevertheless, the fact that self-control was associated with both behaviors almost eight years later highlights its durable role in predicting long-term behavioral outcomes. It is also likely that a variety of unmeasured variables (e.g., externalizing behaviors, substance abuse diagnosis, social modeling effects, *etc.*) explain whether or not sex and alcohol co-occur among young women, lead to compulsive or addictive behavior, and whether or not one behavior may serve as a substitute addiction for the other for some women but not others. There are also some limitations of LCA as an analytic approach. LCA is a statistical technique that relies on mathematical formulations to achieve class assignment. Characterization of the derived classes is “model dependent” and assignment of individuals to their respective classes hinges on the measures used, the sample size, covariates, and interpretational decisions made by the investigator [76]. Thus the derived classes are not literal entities but rather statistical abstractions that contain an element of subjectivity that is imposed on the data [77]. However, LCA methods do help to reduce complexity in behaviors and in the present study have produced important information about the interplay of high-risk drinking and sexual behaviors.

### 4.3. Directions for Future Research

Future studies may expand on the present findings by examining how specific events in the years during the transition to young adulthood influence engagement in risky drinking and sex, as well as the events, experiences, and opportunities that shape the development of self-control and addictive behaviors. These efforts will be important for understanding the nature of these phenomena and for the purposes of developing effect prevention and treatment efforts. This is particularly important because the transition to young adulthood has changed considerably compared to previous generations. The years between adolescence and adulthood are now extended in time with young adults transitioning in and out of school, relationships, jobs, and living arrangements. There are more opportunities for young people to take on different roles and explore various career and lifestyle options and opportunities for self-expression. Adult responsibilities such as marriage, parenting, and career—which have been associated with decreases in risk behavior—occur later now. The extended transition into young adulthood, which now often lasts through the late twenties or beyond, lengthens the period of development when substance use, sexual exploration, and a variety of potentially high risk behaviors have historically peaked in prevalence. It also may provide the time necessary for individuals to develop an extended history with a potentially compulsive behavior, increasing the likelihood of addictive patterns of behavior that interfere with successful development. For these reasons, increasing knowledge about the etiology of risk behaviors during this time of life is increasingly important for effective prevention efforts.
